# Renal arteriolosclerosis impact on clinicopathological features and outcomes of idiopathic membranous nephropathy: a retrospective cohort analysis

**DOI:** 10.3389/fmed.2025.1594990

**Published:** 2025-05-21

**Authors:** Rui Jiang, Li Li, Yunfei Yan, Hucai Li, Ruizhi Yu, Yang Huang, Lixin Wang

**Affiliations:** ^1^The Second Clinical College of Guangzhou University of Chinese Medicine, Guangzhou, China; ^2^Department of Nephrology, The Second Affiliated Hospital of Guangzhou University of Chinese Medicine, Guangzhou, China

**Keywords:** idiopathic membranous nephropathy, arteriolar nephrosclerosis lesion, renal arteriolosclerosis, clinicopathologic, prognosis

## Abstract

**Background:**

To investigate the clinicopathological features and prognostic factors of idiopathic membranous nephropathy (IMN) patients with renal arteriolosclerosis, providing evidence for individualized clinical management.

**Methods:**

A retrospective analysis was conducted on 597 biopsy-confirmed IMN patients at Guangdong Provincial Hospital of Chinese Medicine from January 1, 2012, to December 31, 2022. Patients were stratified into two groups based on the presence of renal arteriolosclerosis. Clinical and pathological characteristics were compared between groups. Kaplan–Meier curves were used to assess cumulative renal remission rates, and Cox regression analysis was performed to identify risk factors for composite endpoint events in IMN patients with arteriolosclerosis.

**Results:**

In a cohort of 597 IMN patients (55.6% male), significant baseline differences were observed in Serum Sodium, triglycerides, membranous nephropathy (MN) stage, mesangial proliferation, interstitial fibrosis, and IgG deposition between the arteriolosclerosis and non-arteriolosclerosis groups (*p* < 0.05). Kaplan–Meier analysis demonstrated markedly lower renal survival in the arteriolosclerosis group (Log-rank χ^2^ = 8.296, *p* = 0.004). Multivariate Cox regression identified age (HR = 1.022, 95% CI 1.003–1.042; *p* = 0.022), serum creatinine (SCr) (HR = 1.010, 95% CI 1.002–1.018; *p* = 0.017), IgM 3 + deposition (HR = 4.718, 95% CI 1.003–1.042; *p* < 0.001), and interstitial fibrosis (HR > 1, *p* < 0.05) as independent risk factors for composite endpoint events, Compared to their respective reference groups, C1q (3+) and tubular atrophy (≥50%) have a protective effect against adverse renal outcomes (HR < 1, *p* < 0.05).

**Conclusion:**

Renal arteriolosclerosis portends poorer prognosis in IMN, with distinct clinicopathological features and accelerated renal function decline. Age, elevated creatinine, intense immune complex deposition, and advanced tubular-interstitial damage represent critical risk markers, highlighting the need for early vascular assessment and histology-guided risk stratification in this population.

## 1 Introduction

Idiopathic membranous nephropathy (IMN) is one of the leading causes of nephrotic syndrome in adults. Over the past two decades, the incidence of IMN has increased approximately 1.5-fold, with a global incidence rate of 1–2 cases per 100,000 person-years. However, the incidence in Asian populations is significantly higher than in Western countries, which may be attributed to genetic predisposition, environmental factors, and improved diagnostic capabilities (1). In China, the rise in IMN prevalence has been particularly pronounced. Epidemiological studies indicate that IMN now accounts for 23.7% of primary glomerular diseases, up from 15.3%, making it one of the most common primary glomerulopathies (2).

Despite advances in PLA2R antibody detection guiding risk stratification, approximately 30–40% of IMN patients progress to end-stage kidney disease within 5–15 years, with relapses occurring in 35–45% of remitted cases under calcineurin inhibitors (3, 4). Current biomarkers fail to predict treatment resistance in 30% of high-titer patients, while comorbidities like diabetes exacerbate therapeutic challenges by impairing glycemic control during immunosuppression (3, 4). Identifying prognostic risk factors for IMN is critical for personalized therapeutic strategies and improving patient outcomes. Furthermore, although histological factors are associated with lower remission rates and more rapid renal function decline, they have not been adequately studied (5). Current research on IMN prognosis has paid limited attention to renal arteriolosclerosis. The clinicopathological characteristics and prognostic implications of IMN patients with concomitant renal arteriolosclerosis remain poorly defined, necessitating further investigation (6, 7).

Therefore, in this study, we retrospectively analyzed the clinicopathological characteristics of patients with IMN complicated by renal arteriolosclerosis, identified factors influencing renal prognosis, and proposed potential management strategies based on these correlations.

## 2 Materials and methods

### 2.1 Study population

**Figure 1:**
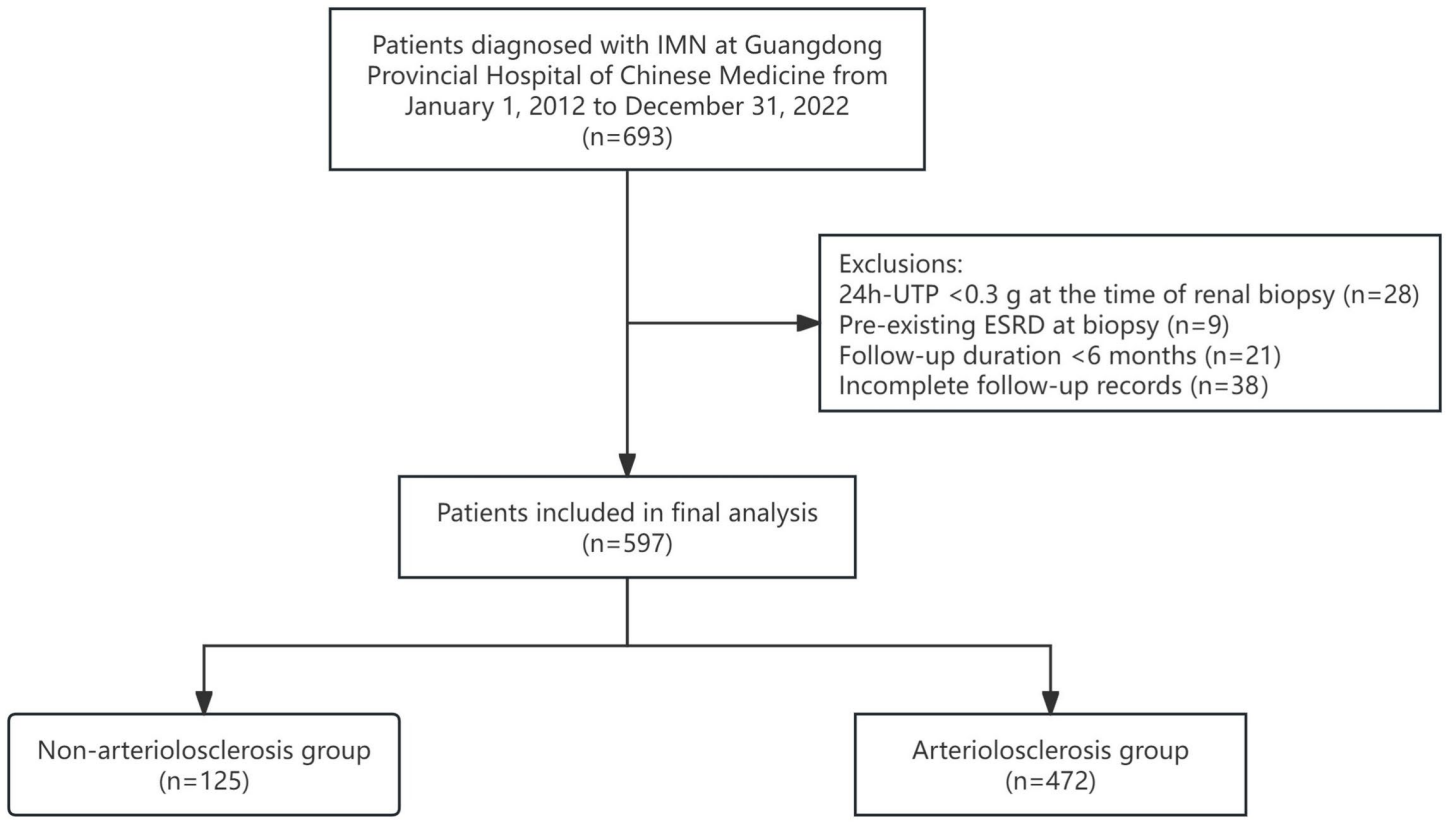
Flowchart of IMN patient selection and arteriolosclerosis-based stratification.

A retrospective analysis was conducted on 597 patients diagnosed with IMN via renal biopsy at Guangdong Provincial Hospital of Chinese Medicine between January 1, 2012, and December 31, 2022 (Figure 1).

#### 2.1.1 Inclusion criteria

(1)  Age ≥18 years, regardless of sex;
(2)  Pathologically confirmed IMN by renal biopsy;
(3)  Complete medical records available.

#### 2.1.2 Exclusion criteria

(1)  Severe psychiatric disorders or other conditions impairing cooperation;
(2)  Clinical diagnosis of secondary membranous nephropathy;
(3)  24-h urinary protein excretion (24 h-UTP) < 0.3 g at the time of renal biopsy;
(4)  Pre-existing End-stage renal disease (ESRD) at biopsy;
(5)  Follow-up duration <6 months.

Patients were categorized into two groups based on the presence of renal arteriolosclerosis: the non-arteriolosclerosis group (*n* = 125) and the arteriolosclerosis group (*n* = 472). This study was approved by the Ethics Committee of Guangdong Provincial Hospital of Chinese Medicine (Ethics approval number: YE 2024-266-01).

### 2.2 Methods

#### 2.2.1 Data collection

Demographic data included sex, age, and comorbidities such as hypertension (HTN), diabetes mellitus (DM), and hyperuricemia (HUA). Laboratory data comprised 24 h-UTP, white blood cell count (WBC), lymphocyte count (LYM), hemoglobin (Hb), platelet count (PLT), blood urea nitrogen (BUN), total carbon dioxide combining rate (TCO_2_), serum uric acid (SUA), serum creatinine (SCr), estimated glomerular filtration rate (eGFR), serum albumin (ALB), triglycerides (TG), total cholesterol (TC), high-density lipoprotein (HDL), low-density lipoprotein (LDL), serum potassium (K^+^), serum sodium (Na^+^), and serum calcium (Ca^2+^). Renal pathological data included findings from light microscopy, electron microscopy, and immunofluorescence studies.

#### 2.2.2 Key definitions

(1)  The follow-up cutoff date was December 31, 2024. A composite endpoint event was defined as any of the following: a ≥ 30% decline in eGFR from baseline, all-cause mortality, progression to ESRD, or failure to achieve partial or complete remission of IMN during follow-up.
(2)  Renal arteriolosclerosis in this study included arteriolar wall thickening, luminal stenosis or occlusion, and hyaline degeneration. Arteriolar wall thickening was assessed according to the criteria established by Zou et al. defined as a ratio of arteriolar internal diameter to external diameter <0.5 (8).
(3)  Complete remission of IMN was defined as stable renal function and 24 h urinary protein < 0.3 g; partial remission is defined as 24 h urinary protein decreased by more than 50% from the baseline (with stable renal function) and < 3.5 g; non—remission is defined as persistent proteinuria with a 24 h urinary protein quantitative reduction < 50% or renal function progression (9).

#### 2.2.3 Statistical analysis

All statistical analyses were performed using R software (version 4.4.2). Continuous variables were tested for normality using the Shapiro–Wilk test. Normally distributed data were expressed as mean ± standard deviation (SD) and compared using independent samples t-tests. Non-normally distributed data were expressed as median (interquartile range, IQR) and compared using the Mann–Whitney U test. Categorical variables were expressed as frequencies and compared using the chi-square test, corrected chi-square test, or Fisher’s exact test, as appropriate.

For prognosis and outcome analysis, Kaplan–Meier curves were plotted, and the log-rank test was used to compare survival rates between groups. Univariate and multivariate Cox regression analyses were performed to identify factors influencing the composite endpoint in IMN patients with renal arteriolosclerosis. Missing data were handled using multiple imputation methods. A two-sided *p*-value <0.05 was considered statistically significant.

## 3 Results

### 3.1 Comparison of baseline characteristics

Among the 597 IMN patients, 265 (44.4%) were female, and 332 (55.6%) were male. Significant differences were observed between the non-arteriolosclerosis and arteriolosclerosis groups only in Na^+^, TG, MN stage, mesangial cell and matrix proliferation, renal interstitial fibrosis, and IgG deposition (*p* < 0.05) (Table 1).

**Table 1:** Comparison of baseline characteristics between groups.

Variables	Non-arteriolosclerosis group (*n* = 125)	Arteriolosclerosis group (*n* = 472)	*χ^2^*/*Z*/*t* value	*p*
Age (years)	53.0 (43.0,62.0)	54.0 (43.0,62.0)	29662.500	0.925
Follow-up time (days)	1200.0 (603.0,2084.0)	1211.5 (601.5,2100.0)	30131.000	0.713
Gender (%)			<0.001	1.000
Male	70 (56.0)	262 (55.5)		
Female	55 (44.0)	210 (44.5)		
HUA (%)			0.128	0.721
No	89 (71.2)	346 (73.3)		
Yes	36 (28.8)	126 (26.7)		
HTN (%)			0.029	0.866
No	84 (67.2)	311 (65.9)		
Yes	41 (32.8)	161 (34.1)		
DM (%)			2.167	0.141
No	103 (82.4)	415 (87.9)		
Yes	22 (17.6)	57 (12.1)		
WBC (×10^9/L)	7.0 (5.7,8.4)	7.0 (5.8,8.4)	29877.500	0.826
LYM (×10^9/L)	2.0 (1.5,2.5)	2.0 (1.6,2.5)	29186.000	0.855
Hb (g/L)	127.7 ± 20.0	128.0 ± 20.4	−0.147	0.883
PLT (×10^9/L)	258.0 (217.0,289.0)	261.0 (217.0,305.0)	27731.000	0.302
24 h-UTP (mg/24 h)	4124.0 (2458.0,6435.0)	4460.0 (2533.3,7207.3)	27660.500	0.283
K^+^ (mmol/L)	4.0 (3.8,4.2)	3.9 (3.7,4.2)	31633.500	0.213
Na^+^ (mmol/L)	140.0 (138.1,142.0)	141.0 (139.0,142.9)	25415.500	0.017*
Ca^2+^(mmol/L)	2.1 (2.0,2.2)	2.1 (2.0,2.2)	30515.500	0.554
SCr (μmol/L)	75.0 (65.0,93.0)	78.0 (58.0,93.2)	30019.500	0.762
eGFR (ml/min/1.73m^2^)	91.4 (74.2,106.2)	89.9 (75.0,105.2)	29950.000	0.793
BUN (mmol/L)	5.0 (4.1,6.5)	5.0 (4.0,6.3)	30735.500	0.471
TCO_2_ (mmol/L)	25.3 (23.4,27.4)	25.3 (23.7,27.5)	28194.500	0.446
SUA (μmol/L)	405.0 (335.0,459.0)	402.0 (336.8,467.2)	29684.000	0.915
ALB (g/L)	25.6 (21.5,30.0)	24.3 (20.8,29.6)	31689.000	0.202
TG (mmol/L)	1.7 (1.0,2.6)	2.1 (1.5,2.8)	24707.500	0.005*
TC (mmol/L)	7.7 (6.2,8.7)	7.6 (6.1,9.4)	28305.000	0.486
HDL (mmol/L)	1.3 (1.1,1.7)	1.3 (1.1,1.7)	28437.500	0.536
LDL (mmol/L)	5.3 (3.8,6.2)	5.1 (3.7,6.9)	29476.000	0.989
MN stage (%)			NA	0.004*
I	6 (4.8)	17 (3.6)		
I-II	23 (18.4)	32 (6.8)		
II	90 (72.0)	390 (82.6)		
II-III	6 (4.8)	31 (6.6)		
III	0 (0.0)	2 (0.4)		
Crescent formation (%)			0.814	0.367
No	119 (95.2)	436 (92.4)		
Yes	6 (4.8)	36 (7.6)		
Mesangial proliferation (%)			31.183	<0.001*
No	16 (12.8)	7 (1.5)		
Yes	109 (87.2)	465 (98.5)		
Endothelial hyperplasia (%)			NA	0.091
No	123 (98.4)	447 (94.7)		
Yes	2 (1.6)	25 (5.3)		
Tubular atrophy (%)			NA	0.075
No	69 (55.2)	198 (41.9)		
<25%	45 (36.0)	218 (46.2)		
≥25% and <50%	9 (7.2)	45 (9.5)		
≥50%	2 (1.6)	11 (2.3)		
Interstitial fibrosis (%)			NA	<0.001*
No	52 (41.6)	91 (19.3)		
<25%	63 (50.4)	316 (66.9)		
≥25% and <50%	7 (5.6)	47 (10.0)		
≥50%	3 (2.4)	18 (3.8)		
Tubular injury (%)			NA	0.050
No	117 (93.6)	401 (85.0)		
Acute	5 (4.0)	46 (9.7)		
Chronic	3 (2.4)	25 (5.3)		
Glomerulosclerosis (%)			0.213	0.645
No	53 (42.4)	187 (39.6)		
Yes	72 (57.6)	285 (60.4)		
IgA (%)			5.724	0.221
−	97 (77.6)	320 (67.8)		
+−	7 (5.6)	28 (5.9)		
+	6 (4.8)	40 (8.5)		
++	9 (7.2)	60 (12.7)		
+++	6 (4.8)	24 (5.1)		
IgG (%)			NA	0.049*
−	2 (1.6)	1 (0.2)		
+−	2 (1.6)	5 (1.1)		
+	3 (2.4)	9 (1.9)		
++	14 (11.2)	27 (5.7)		
+++	104 (83.2)	430 (91.1)		
IgM (%)			NA	0.061
−	78 (62.4)	257 (54.4)		
+−	18 (14.4)	55 (11.7)		
+	22 (17.6)	97 (20.6)		
++	7 (5.6)	48 (10.2)		
+++	0 (0.0)	15 (3.2)		
C3 (%)			NA	0.137
−	5 (4.0)	4 (0.8)		
+−	2 (1.6)	14 (3.0)		
+	6 (4.8)	17 (3.6)		
++	25 (20.0)	97 (20.6)		
+++	87 (69.6)	340 (72.0)		
C1q (%)			7.826	0.098
−	51 (40.8)	139 (29.4)		
+−	15 (12.0)	48 (10.2)		
+	19 (15.2)	88 (18.6)		
++	31 (24.8)	141 (29.9)		
+++	9 (7.2)	56 (11.9)		
FRA (%)			NA	0.403
−	124 (99.2)	459 (97.2)		
+−	0 (0.0)	4 (0.8)		
+	0 (0.0)	7 (1.5)		
++	1 (0.8)	2 (0.4)		

*: *p*<0.05. HUA: Hyperuricemia, HTN: Hypertension, DM: Diabetes Mellitus, WBC: White Blood Cell Count, LYM: Lymphocyte Count, Hb: Hemoglobin, PLT: Platelet Count, 24 h-UTP: 24-h Urinary Protein Excretion, K^+^: Serum Potassium, Na^+^: Serum Sodium, Ca^2+^: Serum Calcium, SCr: Serum Creatinine, eGFR: Estimated Glomerular Filtration Rate, BUN: Blood Urea Nitrogen, TCO_2_: Total Carbon Dioxide Combining Rate, SUA: Serum Uric Acid, ALB: Serum Albumin, TG: Triglycerides, TC: Total Cholesterol, HDL: High-Density Lipoprotein, LDL: Low-Density Lipoprotein, MN: Membranous Nephropathy, Mesangial Proliferation: Mesangial Cell and Matrix Proliferation, Endothelial Hyperplasia: Capillary Endothelial Cell Hyperplasia, Interstitial Fibrosis: Renal Interstitial Fibrosis. NA: Variables were analyzed using Fisher’s exact test.

### 3.2 Analysis of prognostic factors

**Figure 2:**
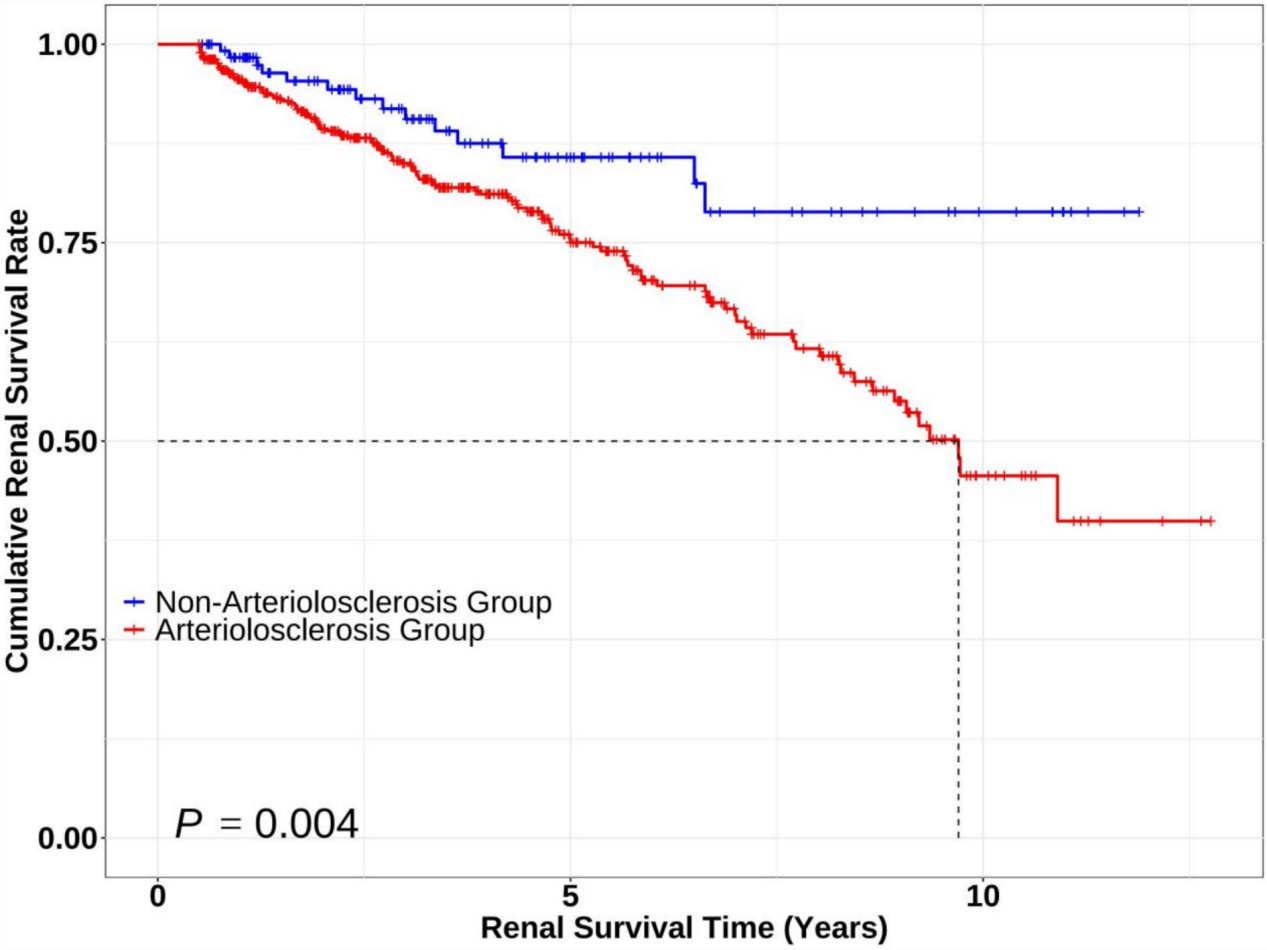
Kaplan–Meier survival curves comparing renal survival between patients with and without arteriolosclerosis. The Kaplan–Meier curves illustrate renal survival probabilities over time for the Non-Arteriolosclerosis Group (*n* = 125, blue line) and Arteriolosclerosis Group (*n* = 472, red line). Statistical significance between groups was assessed using the log-rank test (*p* = 0.004, Log-rank χ^2^ = 8.296).

Kaplan–Meier survival curves were plotted, revealing that the survival curve of the non-arteriolosclerosis group was consistently higher than that of the arteriolosclerosis group throughout the follow-up period. Moreover, the cumulative renal survival rate declined more significantly in the arteriolosclerosis group, with the difference being statistically significant (Log-rank test: *χ*^2^ = 8.296, *p* = 0.004) (Figure 2).

Univariate and multivariate Cox regression analyses were performed on 472 patients with renal arteriolosclerosis. Univariate Cox regression identified sex, age, HUA, HTN, SCr, eGFR, LYM, PLT, BUN, IgM, C1q, tubular atrophy, and renal interstitial fibrosis significantly influenced the prognosis of IMN with renal arteriolosclerosis (*p* < 0.05). Multivariate Cox regression of significant univariate predictors showed that age and SCr were independent risk factors for endpoint events (HR = 1.022, 95% CI 1.003–1.042, *p* = 0.022; HR = 1.010, 95% CI 1.002–1.018, *p* = 0.017). Compared with IgM (−) deposition, IgM (3+) was associated with a higher risk of poor outcomes, whereas C1q (3+) was protective versus C1q (−) (*p* < 0.05). Compared to the no tubular atrophy group, patients with ≥50% tubular atrophy had a significantly decreased likelihood of endpoint events (*p* = 0.039). Compared to no renal interstitial fibrosis, all severity levels of renal interstitial fibrosis were associated with a significantly higher risk of endpoint events (*p* < 0.05) (Table 2).

**Table 2:** Cox regression model analysis of prognostic factors.

	Univariate analysis			Multivariate analysis		
Variables (reference)	*p*	HR	95%CI (lower–upper)	*p*	HR	95%CI (lower–upper)
Gender (male)	<0.001*	0.485	(0.325 ~ 0.724)	0.142	0.713	(0.454 ~ 1.12)
Age	0.001*	1.026	(1.006 ~ 1.012)	0.022*	1.022	(1.003 ~ 1.042)
HUA (no)	0.022*	1.617	(1.073 ~ 2.437)	0.194	1.339	(0.862 ~ 2.082)
HTN (no)	0.004*	1.731	(1.193 ~ 2.512)	0.546	1.138	(0.748 ~ 1.734)
SCr	<0.001*	1.009	(1.006 ~ 1.012)	0.017*	1.010	(1.002 ~ 1.018)
eGFR	<0.001*	0.983	(0.975 ~ 0.990)	0.268	1.010	(0.993 ~ 1.027)
LYM	0.005*	0.696	(0.540 ~ 0.897)	0.060	0.778	(0.600 ~ 1.010)
PLT	0.025*	1.118	(1.013 ~ 1.234)	0.194	1.000	(1.000 ~ 1.001)
BUN	<0.001*	1.135	(1.057 ~ 1.219)	0.681	0.979	(0.886 ~ 1.082)
IgM						
−	Reference			Reference		
+−	0.349	0.735	(0.387 ~ 1.399)	0.825	0.925	(0.462 ~ 1.850)
+	0.141	0.662	(0.382 ~ 1.147)	0.516	0.827	(0.466 ~ 1.467)
++	0.064	1.688	(0.822 ~ 2.430)	0.147	1.572	(0.852 ~ 2.899)
+++	<0.001*	4.164	(1.981 ~ 8.752)	<0.001**	4.718	(2.124 ~ 10.479)
C1q						
−	Reference			Reference		
+−	0.061	0.483	(0.226 ~ 1.033)	0.078	0.487	(0.219 ~ 1.083)
+	0.027*	0.549	(0.323 ~ 0.933)	0.084	0.607	(0.345 ~ 1.069)
++	0.014*	0.548	(0.339 ~ 0.885)	0.050	0.596	(0.355 ~ 1.000)
+++	0.005*	0.387	(0.198 ~ 0.755)	0.018*	0.436	(0.219 ~ 0.870)
Tubular atrophy						
No	Reference			Reference		
<25%	0.021*	1.858	(1.076 ~ 2.462)	0.665	0.900	(0.557 ~ 1.452)
≥25%and<50%	0.076	2.282	(0.942 ~ 3.352)	0.107	0.396	(0.129 ~ 1.220)
≥50%	0.038*	3.125	(1.065 ~ 8.521)	0.039*	0.135	(0.020 ~ 0.907)
Interstitial fibrosis						
No	Reference			Reference		
<25%	0.012*	2.193	(1.192 ~ 4.035)	0.040*	2.080	(1.033 ~ 4.189)
≥25%and<50%	0.004*	3.117	(1.452 ~ 6.691)	0.014*	4.035	(1.319 ~ 12.344)
≥50%	<0.001*	5.529	(2.237 ~ 13.667)	0.005**	12.053	(2.152 ~ 67.510)

*: *p*<0.05, HUA, hyperuricemia; HTN, hypertension; SCr, serum creatinine; eGFR, estimated glomerular filtration rate; LYM, lymphocyte count; PLT, platelet count; BUN, blood urea nitrogen. Interstitial Fibrosis: Renal Interstitial Fibrosis.

### 3.3 Subgroup and sensitivity analyses

**Figure 3:**
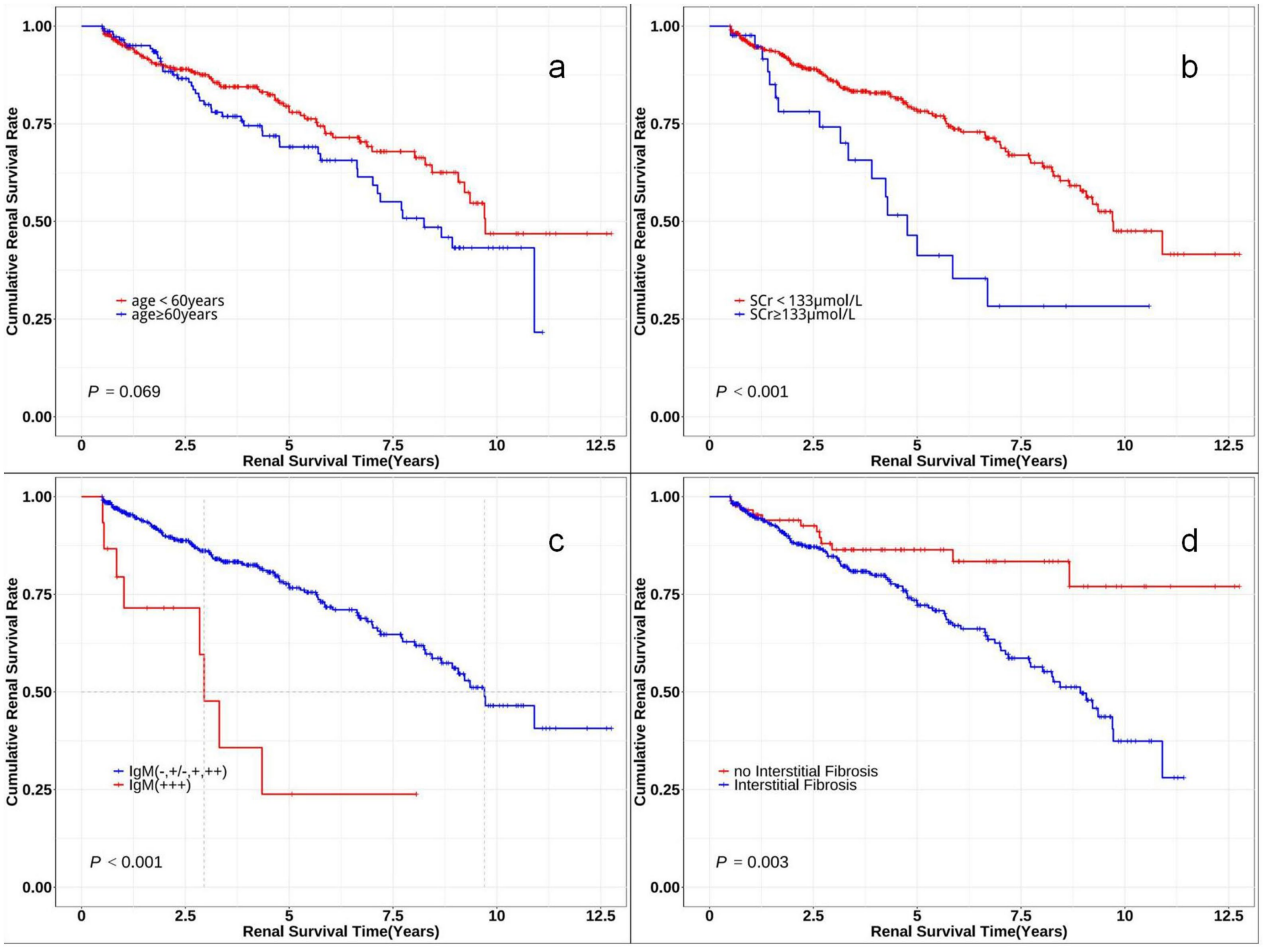
Kaplan–Meier survival curves comparing renal survival of subgroups. The Kaplan–Meier curves for time to renal survival in different subclassifications. **(a)** Probability of renal survival in different age levels (log-rank χ2 = 3.315, *p* = 0.069). **(b)** Probability of renal survival in different serum creatinine (SCr) levels (log-rank χ2 = 13.722, *p* < 0.001). **(c)** Probability of renal survival in different IgM levels (log-rank χ2 = 18.635, *p* < 0.001). **(d)** Probability of renal survival with or without interstitial fibrosis (log-rank χ2 = 8.702, *p* = 0.003).

Figure 3 displays the re-stratified Kaplan–Meier curves, demonstrating lower cumulative renal remission rates in the following subgroups: advanced age cohort (≥60 years), elevated serum creatinine group (SCr ≥ 133 μmol/L), high IgM deposition group (IgM: +++), and patients with renal interstitial fibrosis.

We performed a sensitivity analysis by excluding participants with follow-up duration <1 year, resulting in a final cohort of 404 patients. The refined multivariable Cox proportional hazards model demonstrated comparable results to the primary analysis. Notably, renal interstitial fibrosis lost statistical significance in this long-term follow-up cohort (Table 3).

**Table 3:** Sensitivity analysis of cox model parameters.

Variables (reference)	*p*	HR	95%CI (lower–upper)
Gender (male)	0.171	0.701	(0.422, 1.166)
Age	0.026*	1.024	(1.003, 1.045)
HUA (no)	0.375	1.259	(0.757, 2.095)
HTN (no)	0.808	1.061	(0.660, 1.704)
SCr	0.004*	1.013	(1.004, 1.021)
eGFR	0.260	1.011	(0.992, 1.030)
LYM	0.078	0.771	(0.578, 1.030)
PLT	0.071	1.001	(1.000, 1.001)
BUN	0.699	0.978	(0.876, 1.093)
IgM			
−	Reference		
+−	0.641	1.186	(0.578, 2.433)
+	0.766	0.906	(0.473, 1.736)
++	0.038*	2.015	(1.038, 3.912)
+++	0.005*	4.181	(1.540, 11.352)
C1q			
−	Reference		
+−	0.077	0.452	(0.187, 1.091)
+	0.057	0.551	(0.298, 1.019)
++	0.010*	0.453	(0.249, 0.826)
+++	0.005*	0.316	(0.142, 0.702)
Tubular atrophy			
No	Reference		
<25%	0.993	1.002	(0.583, 1.722)
≥25%and<50%	0.488	0.649	(0.192, 2.198)
≥50%	0.234	0.282	(0.035, 2.271)
Interstitial fibrosis			
No	Reference		
<25%	0.061	2.172	(0.965, 4.889)
≥25%and<50%	0.095	3.011	(0.824, 10.999)
≥50%	0.061	6.531	(0.915, 46.625)

*: *p*<0.05, HUA, hyperuricemia; HTN, hypertension; SCr, serum creatinine; eGFR, estimated glomerular filtration rate; LYM, lymphocyte count; PLT, platelet count; BUN, blood urea nitrogen. Interstitial Fibrosis: Renal Interstitial Fibrosis.

## 4 Discussion

Renal arteriolosclerosis is closely associated with various kidney diseases, and studies have reported its coexistence with hypertensive nephropathy, diabetic nephropathy, IgA nephropathy, chronic kidney disease (CKD) and IMN (10–14). Renal arteriolosclerosis is a vascular lesion characterized by pathological thickening of the arteriolar wall and luminal stenosis, primarily affecting the afferent arterioles and interlobular arteries, leading to abnormal renal hemodynamics and localized ischemic injury (15). Arteriolosclerosis-induced hemodynamic alterations, particularly glomerular hyperfiltration resulting from arteriolar stenosis, enhance mechanical stress on the glomerular basement membrane. This process promotes proteinuria and foot process effacement, which are hallmark features of IMN progression (16). Currently, there is limited research on IMN patients with renal arteriolosclerosis, and most studies have included IMN with vascular lesions or arteriolar intimal thickening as subgroups.

### 4.1 Key findings

In this study of 597 IMN patients (2012–2022), 79% showed concurrent renal arteriolosclerosis, significantly exceeding the previously reported prevalence of 55% (17). The arteriolosclerosis group demonstrated a male predominance (male-to-female ratio: 1.23: 1) and a higher median age compared to the non-arteriolosclerosis group, consistent with the clinical profile of vascular sclerosis susceptibility in elderly males (17, 18). These observations support androgen-mediated vascular inflammatory mechanisms in disease progression (19, 20).

Patients with renal arteriolosclerosis exhibited significantly elevated TG and Na^+^ levels compared to controls, the findings are consistent with those of previous studies (21, 22). A higher proportion of Churg stage II lesions (*p* = 0.003) was observed in this group, likely attributable to hemodynamic disturbances causing metabolic dysregulation of the glomerular basement membrane (23, 24). Prior studies have established associations between renal arteriolosclerosis and renal interstitial fibrosis, mesangial cell proliferation, matrix expansion, and immune complex deposition, which may exacerbate vascular pathology by increasing intraglomerular pressure, oxidative stress, and localized inflammatory responses—consistent with the heightened tubular injury observed in this cohort (24, 25).

After a median follow-up of 1,211.5 days, the arteriolosclerosis group demonstrated a composite endpoint event rate of 23.9%, significantly exceeding the 11.2% rate in the non-arteriolosclerosis group, with reduced renal survival. Multivariate Cox regression analysis identified age, SCr, IgM and C1q (3+) immune deposits, renal interstitial fibrosis, and tubular atrophy ≥50% as independent factors for adverse renal outcomes in IMN patients with arteriolosclerosis.

### 4.2 Mechanistic insights

The pathological interplay between renal arteriolosclerosis and IMN is fundamentally orchestrated by ischemia and hypoxia, a central driver that integrates hemodynamic, oxidative, and metabolic perturbations to amplify renal damage. Specifically, arteriolar luminal stenosis disrupts renal microcirculation, leading to glomerular hyperfiltration and tubulointerstitial ischemia, thereby creating a hypoxic microenvironment that bridges vascular and glomerular injury (26–28). Notably, this hypoxic state triggers hypoxia-inducible factor-1α (HIF-1α) activation, which subsequently upregulates the Wnt/*β*-catenin pathway to promote renal fibrosis through epithelial-mesenchymal transition (EMT) in tubular cells and interstitial fibroblast activation (29).

Moreover, this pathway further exacerbates disease progression by driving renin-angiotensin system (RAS) hyperactivation. As a result, increased angiotensin II production induces arteriolar vasoconstriction and disrupts podocyte slit diaphragms, facilitating subepithelial immune complex retention (30, 31). Importantly, the clinical relevance of this mechanism is strongly supported by evidence demonstrating that RAS blockade with maximum-dose Angiotensin-Converting Enzyme (ACE) inhibitors/Angiotensin II Receptor Blockers (ARBs) achieves comparable 5-year outcomes to immunosuppressive therapy in terms of proteinuria reduction and renal function preservation, as shown by Dikow et al. (32). This finding underscores the dual role of RAS inhibition in mitigating both hemodynamic and non-hemodynamic injury pathways.

Beyond the hypoxia-RAS axis, renal arteriolosclerosis synergistically interacts with IMN through oxidative stress-immune and microbiota-metabolite dysregulation pathways. On one hand, aberrant NOX2/NOX4 activation in sclerotic arterioles generates reactive oxygen species (ROS) that oxidatively modify podocyte PLA2R, exposing cryptic epitopes for IgG4 binding and complement-mediated damage. Additionally, PM2.5-induced extracellular vesicles carrying PLA2R and oxidative stress markers propagate injury through ERK1/2-mediated podocyte cytoskeletal disassembly, establishing a pathogenic “lung-kidney axis” (33). On the other hand, IMN-associated depletion of Lactobacillus and Bifidobacterium species reduces synthesis of AhR-antagonistic indole metabolites, leading to unopposed AhR activation that destabilizes podocytes and recruits inflammatory cells via CXCL16/CXCR6 chemotaxis. Simultaneously, impaired renal clearance of pro-fibrotic metabolites accelerates collagen deposition through myofibroblast activation, creating a self-perpetuating cycle of fibrosis (34). Notably, the identification of C1q (3+) and IgM (3+) deposition as independent factors for adverse renal outcomes prompts mechanistic consideration of how these immune deposits may influence prognosis. We hypothesize that IgM may activate Fc receptors on mesangial cells, inducing pro-inflammatory cytokine release (e.g., IL-6) that could exacerbate arteriolosclerosis-induced ischemia. The apparent paradox of reduced adverse renal outcomes in C1q (3+) compared to no C1q deposition may reflect a threshold effect of complement activation intensity. This potentially indicates more effective regulation of the alternative pathway amplification loop, which becomes critical when classical pathway activation exceeds threshold levels. These hypotheses would require validation through future studies employing electron microscopy for deposit localization, serial measurement of complement split products, and *in vitro* modeling of IgM/C1q-endothelial interactions.

### 4.3 Clinical implications and therapeutic strategies

The coexistence of renal arteriolosclerosis and IMN likely amplifies Cardiovascular Disease (CVD) risk through shared risk factors and cardiorenal interactions. Both conditions are strongly associated with hypertension and dyslipidemia, which are key drivers of atherosclerotic CVD (10). Furthermore, the activation of RAAS in arteriolosclerosis induces myocardial remodeling and sodium retention, while IMN-related hypoalbuminemia and volume overload exacerbate cardiac stress, forming a vicious cycle of cardiorenal syndrome (CRS) with elevated mortality (16, 35).

The prognostic heterogeneity observed across studies regarding renal arteriolosclerosis as a risk factor in IMN stems from three interrelated factors that warrant careful consideration in clinical practice. First, population characteristics play a crucial role, as evidenced by the predominance of elderly patients with advanced vascular fibrosis and multiple comorbidities in our cohort. This demographic profile differs significantly from younger populations in other studies, potentially amplifying ischemia-hypoxia injury and skewing risk estimates (36). Second, inconsistencies in pathological assessment criteria present substantial challenges. The histological spectrum of arteriolosclerosis, ranging from subtle intimal thickening to full-layer hyalinosis, lacks universal diagnostic standards, while variable thresholds for defining severe fibrosis further complicate cross-study comparisons (37). Third, the critical importance of follow-up duration cannot be overstated. Our 3.3-year median follow-up period was necessary to capture the insidious progression of ischemia-driven renal dysfunction, which shorter-term analyses might overlook entirely.

For optimal management of IMN with concurrent arteriolosclerosis, a comprehensive, multimodal therapeutic approach addressing both vascular and immune components is essential. A study of 489 IMN patients showed that the efficacy of immunosuppressive therapy differed among patient groups. Patients <65 years old, female, with proteinuria ≥4.0 g/g or eGFR ≥60 mL/min/1.73 m^2^ had better responses and higher 12-month remission rates, suggesting that renal function and proteinuria levels significantly impact treatment efficacy for IMN patients with renal arteriolosclerosis (38). This could be due to enhanced antigen–antibody reactions in the glomeruli, driven by the impact of arteriolosclerosis on renal microcirculation and subsequent changes in podocyte. The foundation of treatment remains tailored immunosuppressive therapy, with glucocorticoid-cyclophosphamide combinations demonstrating a 65.7% remission rate in experience (36). However, special consideration must be given to elderly patients and those with advanced tubulointerstitial damage (≥30% atrophy), who require careful dose individualization guided by serial PLA2R antibody monitoring and repeat histopathological assessments to balance efficacy against infection risks.

Vascular-protective strategies should be implemented concomitantly. RAS inhibition, particularly with ACE inhibitors or angiotensin receptor blockers, serves dual purposes: reducing proteinuria through intraglomerular pressure modulation while simultaneously countering angiotensin II-induced oxidative stress via NOX4/ROS pathway inhibition (31). Emerging evidence suggests potential adjunctive benefits from antioxidants like N-acetylcysteine, which have shown promising results in preclinical models for attenuating ROS-mediated podocyte injury, though clinical validation studies are still needed (33).

The gut-kidney axis presents novel therapeutic opportunities. Targeted microbiota interventions, including specific probiotic strains like *Lactobacillus plantarum* or dietary modifications to restore aryl hydrocarbon receptor (AhR)-antagonistic bacterial populations, may help modulate tubulointerstitial inflammation. This approach gains credence from preclinical models demonstrating renal protection through fecal microbiota transplantation (39). These strategies should be guided by integrated assessments, which examine renal biopsy findings, particularly arteriolar lesions and immune complex deposition patterns, in conjunction with advanced serological markers.

Regarding lipid management, our analysis of Table 4 revealed several noteworthy findings. Among 473 arteriolosclerosis patients, 152 (32.1%) received statin therapy while only 12 (2.5%) were treated with fibrates, with no cases of familial hyperlipidemia identified. The aggregate 34.7% utilization rate of lipid-lowering medications likely explains the lack of significant association between lipid parameters and composite endpoints in Cox regression analysis. This suggests that contemporary lipid management protocols may be effectively maintaining lipid levels below nephrotoxic thresholds in approximately one-third of patients. However, the potential benefits of more aggressive lipid control in high-risk subgroups—particularly those with extensive vascular pathology or progressive renal dysfunction—merit further investigation through prospective studies with protocolized treatment algorithms and longer follow-up periods. These findings underscore the importance of individualized risk stratification when considering therapeutic intensity for dyslipidemia in this complex patient population.

**Table 4:** Cox regression model analysis of lipid profile results.

Variables (reference)	*p*	HR	95%CI (lower–upper)
Triglyceride	0.104	1.079	(0.984 ~ 1.184)
Total cholesterol	0.315	0.964	(0.897 ~ 1.035)
High—density lipoprotein	0.474	0.94	(0.793 ~ 1.114)
Low—density lipoprotein	0.753	0.988	(0.917 ~ 1.095)

### 4.4 Limitations

This study has several limitations. First, as a single-center retrospective study with a population from a geographically limited area, the findings may not be generalizable to all ethnic groups. Second, due to the retrospective nature of the study, some early-stage patients lacked PLA2R antibody testing results and certain serological immunological markers at the time of renal biopsy, and were therefore excluded. Finally, data regarding renal artery atherosclerosis were not collected due to the inherent limitations of retrospective study design. We look forward to future multicenter, large-cohort studies to further explore the relationship between renal arteriolosclerosis and IMN.

## 5 Conclusion

This study demonstrates that renal arteriolosclerosis is a critical determinant of adverse outcomes in patients with IMN, characterized by distinct clinicopathological features and accelerated renal function decline. In a cohort of 597 biopsy-confirmed IMN patients, those with arteriolosclerosis exhibited significantly elevated Na^+^, TG, advanced MN stages, mesangial proliferation, interstitial fibrosis, and IgG deposition compared to non-arteriolosclerosis counterparts. Kaplan–Meier analysis revealed markedly reduced renal survival in the arteriolosclerosis group (log-rank *χ*^2^ = 8.296, *p* = 0.004), with a composite endpoint event rate of 23.9% versus 11.2% in controls. Multivariate Cox regression identified age, elevated SCr, intense IgM (3+) deposition, and interstitial fibrosis as independent risk factors for adverse renal outcomes. Conversely, C1q (3+) deposition and tubular atrophy (≥50%) demonstrated protective effects, suggesting a complex interplay between complement activation thresholds and tubular adaptive responses.

These findings underscore the necessity of integrating vascular assessment into the diagnostic and prognostic evaluation of IMN. Early identification of arteriolosclerosis, coupled with histology-guided risk stratification, may enable personalized therapeutic strategies targeting both immune-mediated injury and vascular pathology. Clinicians should prioritize aggressive management of modifiable risk factors, such as optimizing blood pressure control via RAS inhibition and mitigating oxidative stress, while tailoring immunosuppressive regimens based on histological severity. Future multicenter studies are warranted to validate these associations, explore mechanistic links between complement deposition and vascular remodeling, and evaluate the efficacy of novel interventions, including microbiota modulation and targeted antifibrotic therapies, in this high-risk population.

## Data Availability

The original contributions presented in the study are included in the article/supplementary material, further inquiries can be directed to the corresponding author.
